# Effusive-constrictive pericarditis, hepatitis, and pancreatitis in a patient with possible coxsackievirus B infection: a case report

**DOI:** 10.1186/s12879-016-1752-3

**Published:** 2016-08-08

**Authors:** Jon Persichino, Roger Garrison, Rajagopal Krishnan, Made Sutjita

**Affiliations:** 1 Department of Internal Medicine, Riverside University Health System Medical Center, 26520 Cactus Avenue, Moreno Valley, CA 92555 USA; 2 Division of Cardiology, Riverside University Health System Medical Center, Moreno Valley, CA USA; 3 Division of Infectious Diseases, Riverside University Health System Medical Center, Moreno Valley, CA USA

**Keywords:** Coxsackie B, Effusive-constrictive pericarditis, Hepatitis, Pancreatitis

## Abstract

**Background:**

Coxsackie B is a viral pathogen that presents with various invasive diseases in adults. Historically, the majority of adult cases with pericarditis or myocarditis have been attributed to coxsackievirus B. The presentation of this viral infection causing effusive-constrictive pericarditis, hepatitis or pancreatitis is rare. This case report is the first to describe a patient with concomitant effusive-constrictive pericarditis, hepatitis and pancreatitis from possible coxsackievirus B infection.

**Case presentation:**

A 26-year old female was admitted to our hospital with the diagnosis of effusive-constrictive pericarditis complicated by tamponade and cardiac arrest. An emergent pericardiocentesis was performed successfully. Hepatitis and pancreatitis were also identified in our patient. After an extensive workup, coxsackievirus B infection was suspected by positive serum complement fixation antibody titers. Our patient made a full recovery and was discharged from the hospital.

**Conclusion:**

Clinical suspicion of effusive-constrictive pericarditis with tamponade from coxsackievirus B should be considered in patients presenting with chest pain, dyspnea, jugular venous distention, hypotension, ST segment elevation on electrocardiogram, and ventricular interdependence with septal shift during diastole on transthoracic echocardiogram. Initial diagnoses of effusive-constrictive pericarditis resembling cardiac tamponade, hepatitis and pancreatitis can be challenging, and this case highlights the need for healthcare professionals to be cognizant of the association between these unusual clinical presentations and coxsackievirus B infection.

**Electronic supplementary material:**

The online version of this article (doi:10.1186/s12879-016-1752-3) contains supplementary material, which is available to authorized users.

## Background

Coxsackievirus, a RNA *Enterovirus*, has been traditionally associated with a number of clinical diseases in children and adults. The species is divided into two groups and 29 serotypes [1]. Coxsackie group A viruses can cause aseptic meningitis in adults, and commonly infect skin and mucous membranes in herpangina, conjunctivitis and hand, foot and mouth disease in children [1]. Group B viruses cause herpangina, pleurodynia, and infect the heart, pancreas, and liver which can give rise to myocarditis, pericarditis, pancreatitis and hepatitis in adults [2]. Viral pericarditis is inflammation of the pericardium or the lining surrounding the heart that is caused by viral infections [2]. Coxsackie B viruses are the most common cause of myocarditis and pericarditis in adults and have been identified in up to 50 % of viral cardiac cases [3–5]. A number of other viral, bacterial, and fungal infections as well as medications has also been shown to cause myocarditis and pericarditis [2, 6]. However, the presentation of this viral infection is rare among patients with isolated pancreatitis or hepatitis [7–11]. Up to 60 % of patients with acute pericarditis will develop a small pericardial effusion [6]. Notably, viral pericarditis can lead to pericardial constriction as a late complication [12, 13]. Constrictive pericarditis with effusion has been detected in ten percent of patients with clinical tamponade [2]. Coxsackie B viruses usually cause various single organ system diseases, but the combination of myocarditis, pancreatitis and hepatitis has been documented in two case reports [14, 15]. Herein, we report a case of possible coxsackievirus B infection causing effusive-constrictive pericarditis, hepatitis and pancreatitis.

## Case presentation

A 26-year old Latina female sought medical attention at our emergency department with progressive throat pain and chest pain for 1 week. She then developed fevers, dizziness, shortness of breath, and abdominal pain with nausea and vomiting 2 days prior to admission. She did not seek prior medical attention and used over-the-counter ibuprofen for the fevers. The patient denied any recent travel, unusual food consumption or animal exposures.

Further history revealed that the patient’s 10-year old sister was recovering from an upper respiratory infection with fevers, throat pain, cough and shortness of breath for 1 week. Past medical history was significant for congenital adrenal hyperplasia. Our patient denied current usage of tobacco, alcohol, or intravenous drugs. She had no known drug allergies and had been taking physiologic doses of hydrocortisone 30 milligrams (mg) per day and fludrocortisone 0.5 mg per day for her congenital adrenal hyperplasia. The patient disclosed to the medical team that she had run out of her hydrocortisone and fludrocortisone approximately 4 to 6 weeks prior to admission.

**Fig. 1:**
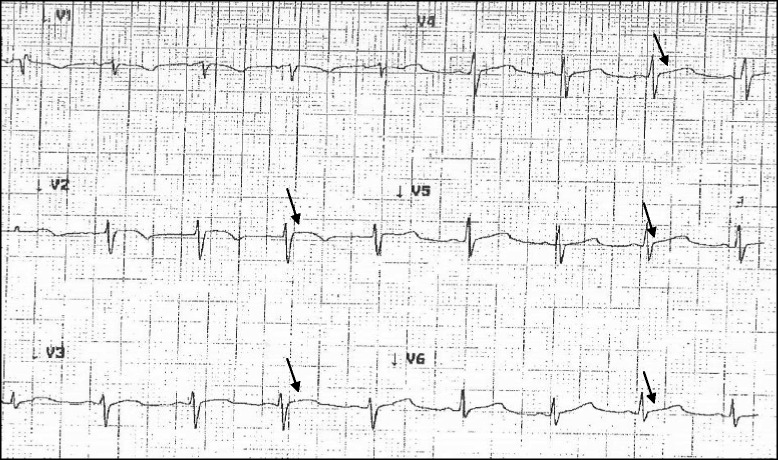
Electrocardiogram from patient with ST-segment elevations (arrows) in V2-V4 precordial leads

Initial vital signs in the emergency department demonstrated a temperature of 102.0 °F (38.9 °C), heart rate of 102 per minute, respiratory rate of 28 per minute, oxygen saturation of 95 % while breathing 6 liters of oxygen per minute via face mask, and blood pressure of 50/21 millimeters of mercury. On examination, she was ill appearing and diaphoretic. There was jugular venous distention by visualization of the neck veins and distant heart sounds upon auscultation. Aggressive intravenous fluid hydration, vasopressor medications, and intravenous hydrocortisone were initiated for suspected septic shock and adrenal crisis. Supplemental oxygen was provided by face mask. Empiric antibiotic treatments with vancomycin, levofloxacin, fluconazole, and metronidazole were initiated based upon consultation with the infectious diseases specialist. Blood and urine cultures were collected in the emergency department before antibiotic administration. Pertinent laboratory findings in our patient on admission and during hospitalization are shown on Table 1. A 12-lead electrocardiogram (ECG) showed 1–2 mm ST segment elevations in precordial V2-V6 leads as shown in Fig. 1. Pericarditis with tamponade was suspected by the emergency room personnel. An emergent bedside transthoracic echocardiogram (TTE) was performed by a technician which revealed a small pericardial effusion. A portable chest radiograph was remarkable for vascular congestion only. Two hours after her arrival to the emergency department, the patient developed cardiac arrest. Adult cardiac life support was initiated in which intubation and cardiopulmonary resuscitation were performed. Intravenous epinephrine was given for pulseless electrical activity. Pulse was restored after three minutes of treatment. An emergent bedside pericardiocentesis with drain placement was carried out by the on-call cardiologist. Three-hundred milliliters of serosanginous pericardial fluid were extracted. A repeat bedside TTE revealed minimal residual effusion of the pericardium with normal ventricular function. The patient was admitted to the intensive care unit with the diagnosis of effusive-constrictive pericarditis complicated by tamponade, cardiac arrest and adrenal crisis.

**Table 1:** Laboratory values in our patient on admission, during hospitalization, and post-hospital follow-up

	Normal value	Day 1	Day 4	Day 8	Day 12	Day 16	Follow-up After 4 weeks
TB	0.2–1.0 mg/dl	2.3	1.4	0.6	0.4	0.4	0.6
ALT	12–78 U/L	109	868	420	138	86	34
AST	15–37 U/L	213	1,060	189	21	22	29
CK	26–192 U/L	225	353	521	NR	NR	NR
CKMB	0.5–3.6 ng/ml	3.4	8.4	14.0	NR	NR	NR
Troponin	0.000–0.045 ng/ml	0.44	0.89	0.45	NR	NR	NR
Amylase	25–115 U/ml	102	212	NR	NR	NR	NR
Lipase	73–393 U/L	773	1,739	7,880	4,850	NR	262

*TB* total bilirubin, *mg* milligrams, *dl* deciliter, *ALT* alanine aminotransferase, *U* units, *L* liter, *AST* aspartate transaminase, *CK* creatinine kinase, *CKMB* creatinine kinase MB, *ng* nanograms, *ml* milliliter, *NR* no result

Additional samples of blood and urine were collected for screening of bacterial (*Streptococcus*, *Staphylococcus*, Meningococcus, *Haemophilus*, *Legionella*), fungal (*Histoplasma*, *Aspergillus*, *Candida*, *Cryptococcus*, Coccidiomycosis), viral (herpesviruses, human immunodeficiency virus, Epstein-Barr virus, hepatitis A, B, and C, Cytomegalovirus), and mycobacterial (*Mycobacterium tuberculosis*, Mycobacterium avium complex) infections with negative results. Serological paired complement fixation antibody titers for coxsackie A and B were sent on days 1 and 7 of hospitalization. A panel of serological tests for rheumatologic, autoimmune and malignant diseases were performed as well. All pericardial fluid stains and cultures were negative for bacterial, fungal and mycobacterial diseases. Rheumatologic, autoimmune and malignancy workups were negative. Our infectious disease specialist suspected coxsackievirus infection given patient’s presentation and history of recent sick contact with her younger sister. Coxsackie titer antibodies were negative for group A serotypes A2, A4, A7, A9, A10 and A16. Coxsackie B titer antibodies on day 1 were: B1 (<1:8), B2 (1:16), B3 (1:8), B4 (<1:8), B5 (1:16), and B6 (1:16). Coxsackie B titers on day 7 of hospitalization were: B1 (1:8), B2 (1:16), B3 (1:8), B4 (1:8), B5 (1:16), and B6 (1:16). Real-time polymerase chain reaction (PCR) of pericardial fluid for enteroviruses was sent on admission but our outside reference laboratory did not have the capability in performing the test. Viruses were not isolated on the viral culture of the pericardial fluid. With continued supportive medical care for the complications following suspected coxsackievirus B infection, our patient made a full recovery after 17 days in the hospital. Five months after her hospitalization, a repeat TTE showed normal left ventricular function and pericardium in our patient.

## Discussion

To the best of our knowledge, this is the only case report of a patient with concomitant effusive-constrictive pericarditis, hepatitis and pancreatitis described in the medical literature. Coxsackie B 1, 2, and 6 group serotypes can be associated with complicated disease with left ventricular dysfunction and myocardial infarction from myopericarditis and acute liver failure from hepatitis [4, 5, 15]. Diagnosis is largely based on clinical manifestations of disease with positive serological tests. In the acute setting of her rising, positive antibody titers and clinical presentation of disease, we believe that our patient acquired coxsackievirus B from her sick, younger sister. Serological markers for coxsackie B virus have been associated with the development of myocarditis and pericarditis [5]. The combination of myocarditis, hepatitis and pancreatitis due to coxsackie B infection is very rare. Two such reported cases were attributed to coxsackie A4 and B2 serotypes [14, 15]. The disease process of acute pericarditis can progress to a large pericardial effusion that can cause cardiac tamponade. Our case differed from classic cardiac tamponade in that there was not a large pericardial effusion but a stiff pericardium. Only one of the prior published cases reported a presentation of isolated effusive-constrictive pericarditis by coxsackie A4 and B3 [13]. There are few case reports on hepatitis associated with coxsackie virus infection [5, 7, 8]. In a retrospective study of 602 patients with acute pancreatitis, 22.1 % of cases were probably attributed to coxsackie viral infection [11]. In a separate study, only 4.3 % of patients with acute pancreatitis exhibited significant rising antibody titers to coxsackie B virus [11]. The combination of myocarditis and pancreatitis from coxsackievirus is few and far between given that the rate of asymptomatic pancreatitis may be higher than previously thought in infected patients with myocardial involvement [14].

Acute pericarditis is a rare phenomenon that accounts for only 0.2 % of all hospital cardiovascular admissions [6]. Approximately 80–90 % of pericarditis cases in North America and Western Europe are presumed to be caused by enteroviruses, herpesviruses, and adenoviruses [6]. Diagnosis of acute pericarditis can be confirmed by chest pain that worsens in the recumbent position, a friction rub upon auscultation, and diffuse ST segment elevation on the ECG. These characteristic changes on the ECG can be seen in approximately 50 % of patients with acute pericarditis [2]. Sixty percent of cases with acute pericarditis also present with a small pericardial effusion [6].

Rarely, acute pericarditis can give rise to a large pericardial effusion that can cause tamponade. Reduced QRS voltage and electrical alternans can be displayed on the ECG with a large pericardial effusion. Cardiac tamponade presents with dyspnea, jugular venous distention and hypotension. Jugular venous distension is the most common physical finding in acute cardiac tamponade [2]. Cardiac tamponade decreases ventricular diastolic filling which results in reduction of stroke volume and cardiac output. The most useful tool for the diagnosis of pericarditis and pericardial effusion is the TTE. Pericardiocentesis is the treatment of choice for patients presenting with cardiac tamponade. Viruses are rarely isolated from pericardial fluid after pericardiocentesis [2]. Pericardiotomy with biopsy and drainage produce the greatest diagnostic yield for viral identification [2]. In patients with persistent constriction after pericardiocentesis, extensive pericardiectomy is recommended [12].

**Fig. 2:**
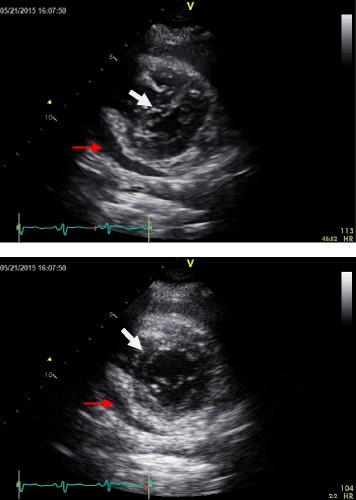
Parasternal short axis still frames of echocardiogram from patient demonstrating small pericardial effusion (red arrows) with ventricular interdependence for diastolic filling as evidenced by a septal shift to the left during right ventricular filling (upper panel, white arrow) and septal shift to the right during left ventricular filling (lower panel, white arrow). In the setting of shock and pericardial effusion, septal bounce is pathognomonic for effusive-constrictive pericarditis with cardiac tamponade

Acute pericarditis can also lead to constriction as a late complication [2]. Effusive-constrictive pericarditis accounts for 10–20 % of patients with clinical tamponade by reducing ventricular diastolic filling due to a stiff pericardium [2, 12]. It is an uncommon syndrome that may be missed in some patients who present with tamponade. A large prospective case review revealed that concomitant constriction with tamponade was recognized in only 7 out of 1,184 patients [12]. As illustrated in Fig. 2 and Additional file 1, the initial TTE of our patient demonstrated a small pericardial effusion with ventricular interdependence for diastolic filling and septal bounce. This unique and rare occurrence on TTE is highly indicative of effusive-constrictive pericarditis with cardiac tamponade.

Pericarditis and myocarditis may coexist in up to 30 % of infectious and non-infectious cases [6]. Most cases of viral myocarditis are caused by coxsackie B serotypes [14]. Myocarditis can markedly increase troponin levels in the serum. There was no evidence to suggest that our patient had myocarditis. The small rise of troponin levels in her serum was believed to have been caused by demand ischemia from reduced cardiac output due to tamponade as well as cardiopulmonary resuscitation and reduced clearance from acute renal failure.

The most effective treatment for viral pericarditis is nonsteroidal anti-inflammatory drugs (NSAIDs) [2, 6]. Colchicine can be used in combination with NSAIDs to further decrease recurrence rates [2, 6]. The use of low dose corticosteroids can also be used for treatment and to lower the recurrence rate in patients who do not respond, are intolerant, or have contraindications to NSAIDs and colchicine [2, 6]. We did not treat our patient with NSAIDs and colchicine due to acute renal failure and possible increased gastrointestinal intolerance if used together with her stress doses of corticosteroids.

Our case report has several limitations. First, our patient’s complement fixation antibody titers for coxsackie B were not significantly elevated during the early course of infection. We believe that titers of 1:8 to 1:16 may be indicative, but not confirmative, of recent infection. There is also considerable cross-reactivity between antibody titers among other enteroviruses. Second, since titer levels persist for a few months, we did not send a convalescent specimen to determine if there was a significant rise in titers to confirm our diagnosis. Third, we were unable to confirm coxsackie B infection by PCR detection. This case report can highlight the importance for all outside reference laboratories to obtain the capability in performing real-time PCR of pericardial fluid specimens for enteroviruses. In our patient, based on her clinical presentation of pericardial, hepatic, and pancreatic disease with rising, positive antibody titers, we believe that coxsackievirus B infection was the likely cause.

## Conclusions

This case report describes a rare presentation of possible coxsackievirus B infection causing sequential effusive-constrictive pericarditis, hepatitis, and pancreatitis in a patient following recent exposure. Medical professionals need to be aware that coxsackievirus B can lead to effusive-constrictive pericarditis resulting in the development of cardiac tamponade and cardiac arrest if left untreated. This case illustrates that rapid accumulation of a small amount of pericardial fluid can result in tamponade physiology. Clinical suspicion of effusive-constrictive pericarditis should be considered in patients presenting with chest pain, jugular venous distention, ST segment elevation on the ECG, hypotension and small pericardial effusion on TTE. Particularly in patients with signs and symptoms of tamponade, clinicians should be vigilant in initiating emergent treatment with pericardiocentesis. With better awareness and early diagnostic and invasive interventions, clinicians can make a substantial difference in the care of their patients with complications from coxsackievirus B infection.

## Abbreviations

dl, deciliter; ECG, electrocardiogram; L, liter; mg, milligrams; ml, milliliter; ng, nanograms; NSAIDs, nonsteroidal anti-inflammatory drugs; PCR, polymerase chain reaction; RNA, ribonucleic acid; TTE, transthoracic echocardiogram; U, units

## Additional file

Additional file 1: Echocardiogram of patient showing small pericardial effusion with ventricular interdependence for diastolic filling as evidenced by septal bounce due to effusive-constrictive pericarditis and cardiac tamponade. (MOV 158 kb)
